# Immune–Inflammatory Hub Genes Intersecting with a Ferroptosis-Associated Gene Set in Active Tuberculosis: A Multi-Dataset Bioinformatics Study

**DOI:** 10.3390/ijms27177757

**Published:** 2026-08-29

**Authors:** Rasha Elsayim, Monerah S. M. Alqahtani, Malek Hassan Ibrahim Alaaullah, Reem A. Bin Suaydan, Esra’a Abudouleh, Sami Habiballa Abdalla Mohamed, Nihal Almuraikhi

**Affiliations:** 1 Department of Botany and Microbiology, College of Science, King Saud University, P.O. Box 2455, Riyadh 11451, Saudi Arabia; 2 Biology Department, Faculty of Science, King Khalid University, Abha 61413, Saudi Arabia; 3 Disaster and Emergency Medicine, Brown University, Providence, RI 02912, USA; 4 Department of Clinical Laboratory Science, College of Applied Medical Sciences, King Saud University, Riyadh 11451, Saudi Arabia; 5 Department of Health Sciences, College of Applied Studies, King Saud University, Riyadh 11451, Saudi Arabia; 6 Department of Anatomy, College of Medicine, King Saud University, Riyadh 11451, Saudi Arabia

**Keywords:** ferroptosis, active tuberculosis, latent tuberculosis, *Mycobacterium tuberculosis*, multi-omics analysis

## Abstract

The progression of tuberculosis (TB) from a latent infection to an active disease involves intricate modifications in host immune, inflammatory, oxidative, and metabolic pathways. Ferroptosis represents a distinct form of regulated cell death that requires iron and is associated with excessive lipid peroxidation and has been associated with tissue damage in TB; however, its connection with host transcriptional changes during active TB is not fully understood. This study sought to identify and externally validate immune–inflammatory hub genes among differentially expressed genes (DEGs) in active TB that overlap with a ferroptosis-associated gene set derived from FerrDb, employing an integrated transcriptomic and systems-biology methodology. Differential expression analysis of GSE37250 revealed 1015 DEGs in active TB compared to latent TB, comprising 585 upregulated and 430 downregulated genes, and 93 DEGs in active TB compared to healthy controls, including 65 upregulated and 28 downregulated genes. Intersection analysis identified 94 DEGs common to the active TB versus latent TB comparison and the ferroptosis-associated gene set, and eight DEGs common to the active tuberculosis versus healthy-control comparison and the same gene set, with no genes shared across all three sets. Functional enrichment of the 94 intersection genes underscored immune response, defense response, stress response, Toll-like receptor signaling, NOD-like receptor signaling, IL-17 signaling, TNF signaling, glutathione metabolism, neutrophil degranulation, cytokine signaling, and antimicrobial metal sequestration. Protein–protein interaction analysis followed by cytoHubba prioritization identified 10 hub genes: IL1B, TLR4, CXCL10, MMP9, CYBB, MPO, CD36, LCN2, S100A8, and LTF. Subsequent to outcome-independent probe selection, external validation in GSE28623 demonstrated significant positive differential expression of LCN2, S100A8, and LTF, while GSE62525 showed significant positive differential expression of IL1B, TLR4, MMP9, MPO, LCN2, and LTF. LCN2 and LTF were significantly upregulated in both validation datasets, indicating the strongest cross-dataset reproducibility. These results identify an immune–inflammatory transcriptional network intersecting with ferroptosis-associated genes in active TB. Notably, the transcriptomic findings do not confirm ferroptotic cell death but suggest candidate genes and biological processes for future experimental exploration.

## 1. Introduction

Tuberculosis (TB) is an infectious disease caused by *M. tuberculosis* and continues to be a significant infectious disease globally. Despite progress in diagnostic and therapeutic methods, TB still results in considerable illness and death. The World Health Organization reports that in 2024, TB claimed the lives of 1.23 million individuals, including 150,000 people with HIV, and remains a leading cause of death linked to antimicrobial resistance [1]. Approximately one-quarter of the global population is infected with *M. tuberculosis*; however, only a portion of these individuals transition from latent tuberculosis infection (LTBI) to active disease. This highlights the clinical significance of understanding the biological determinants of disease progression.

The shift from latent infection to active TB is influenced by intricate interactions between *M. tuberculosis* and the host’s immune system. Active TB is linked to disrupted inflammatory responses, the activation of macrophages, the recruitment of neutrophils, oxidative stress, and immune pathology that damages tissues. Whole-blood transcriptomic studies have consistently identified signatures associated with interferon and neutrophils in active tuberculosis (TB). These signatures have demonstrated the capability to differentiate active disease from latent TB infection (LTBI) and other clinical conditions [2,3,4,5]. These studies confirm that TB progression involves significant host transcriptional changes, yet the molecular mechanisms connecting immune activation, oxidative damage, lipid dysregulation, and regulated cell death during active TB are not clearly established. Ferroptosis is an iron-dependent mechanism of regulated cellular death that is iron-dependent and non-apoptotic, mediated by lipid peroxidation and elevated levels of reactive oxygen species. The relevance of these processes to tuberculosis (TB) lies in the fact that Mycobacterium tuberculosis infection disrupts host iron homeostasis, induces oxidative stress, and leads to macrophage damage and tissue necrosis.

Experimental data suggest that ferroptosis may contribute to the pathogenesis of tuberculosis (TB). Amaral et al. demonstrated that cell death in macrophages infected with Mycobacterium tuberculosis depends on iron-mediated oxidative damage to cellular lipids. They further showed that inhibiting ferroptosis decreased cell death and tissue damage [6]. Additional research has implicated changes in iron homeostasis, oxidative stress, and antioxidant defenses in mycobacterial infection [7]. Recent bioinformatics studies have also identified ferroptosis-associated candidate signatures in TB, including ACSL4, CTSB, TLR4, ACSL1, PARP9, and ATG3 [8,9]. The available evidence emphasizes the contribution of ferroptosis-related genes in TB diagnosis and pathogenesis; however, the field is constrained by variations in dataset selection, gene prioritization methods, validation depth, and the biological interpretation of ferroptosis-related immune–inflammatory networks. Despite these developments, a significant research gap persists. Most prior TB transcriptomic studies have broadly focused on interferon signaling, immune activation, or general biomarker discovery, with fewer studies specifically integrating ferroptosis-related genes with differential gene-expression profiling, pathway and functional annotation, protein-interaction network construction, hub-gene selection and evaluation in independent datasets, and mechanistic interpretation. Particularly, the connection between ferroptosis-related transcriptional changes and key immune processes such as Toll-like receptor signaling, neutrophil degranulation, reactive oxygen species production, lipid dysregulation, antimicrobial metal sequestration, and tissue remodeling in active TB remains inadequately understood. We therefore investigated whether immune–inflammatory hub genes emerging from the ferroptosis-associated DEG intersection show reproducible expression patterns across independent active TB versus latent TB transcriptomic datasets. Consequently, the study aimed to identify and validate immune–inflammatory hub genes detected within the ferroptosis-associated DEG intersection that are associated with active TB progression through an integrated bioinformatics approach. We employed a comprehensive approach to identify immune–inflammatory candidates associated with active tuberculosis. This approach integrated differential-expression analysis, the intersection of a ferroptosis-associated gene set derived from FerrDb, functional enrichment, network prioritization, and external transcriptomic validation. This study builds upon prior research by investigating the intersection of transcriptional changes in active tuberculosis with a curated set of genes associated with ferroptosis. Additionally, it assesses network-prioritized candidates across independent datasets.

## 2. Results

### 2.1. Gene-Expression Profiling Demonstrated Unique Transcriptional Alterations in Active TB

**Figure 1:**
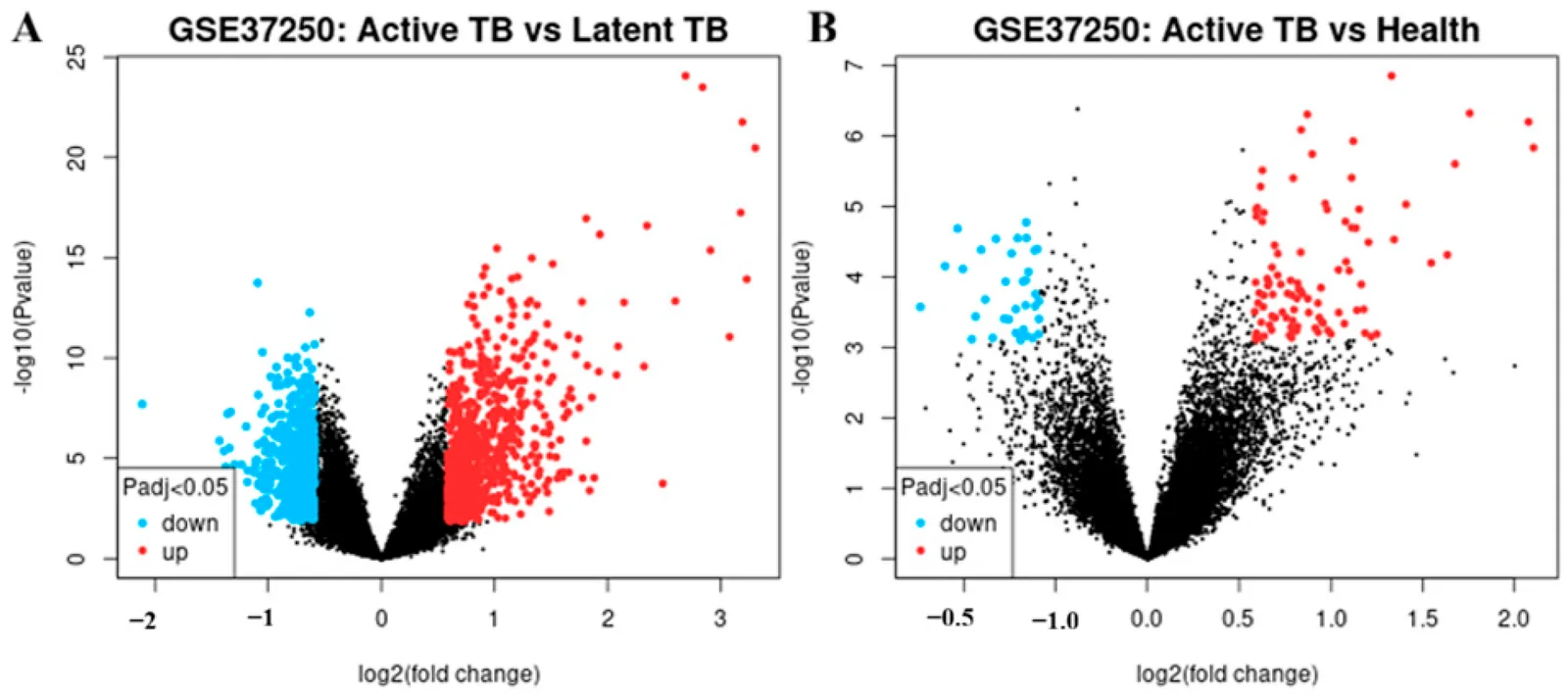
Differential expression analysis of GSE37250. (**A**) Volcano plot showing differentially expressed genes in active TB versus latent TB. (**B**) Volcano plot showing differentially expressed genes in active TB versus healthy controls. Red dots indicate significantly upregulated genes, blue dots indicate significantly downregulated genes, and black dots represent nonsignificant genes. Differential expression was defined using an adjusted *p* < 0.05. The active TB versus latent TB comparison showed a broader transcriptional shift than the active TB versus healthy control comparison.

Using GSE37250, differential expression analysis was conducted to contrast active tuberculosis (active TB) with latent tuberculosis infection (latent TB) and healthy individuals. Volcano plots were utilized to depict genes that were significantly upregulated or downregulated; genes with *p*adj < 0.05 were considered statistically significant. The comparison between active TB and latent TB exhibited a more extensive transcriptional variation than that between active TB and healthy controls. In total, 1015 differentially expressed genes (DEGs) were found when comparing active TB to latent TB, comprising 585 upregulated and 430 downregulated genes. Notably upregulated genes included GBP6, FCGR1A, ANKRD22, CACNA1E, and FCGR1B, while FCGBP, ID3, LRRN3, LINC00926, and MS4A1 were among the most downregulated. In the comparison of active TB with healthy controls, 93 DEGs were identified, with 65 genes upregulated and 28 downregulated. Prominent upregulated genes were SERPING1, GBP6, ANKRD22, GRAMD1B, and GBP5, whereas MS4A1, RPS6KA3, ZNF608, BANK1, and GPCPD1 were notably downregulated. Overall, the current evidence suggests that active TB is linked to significant transcriptional changes, with the most pronounced differential expression seen in the active tuberculosis versus latent TB comparison. This underscores the importance of using the active disease versus latent TB comparison for the main analysis to discover genes related to TB progression, as shown in Figure 1A,B.

### 2.2. Identification of 94 DEGs Intersecting with the FerrDb-Derived Ferroptosis-Associated Gene Set

**Figure 2:**
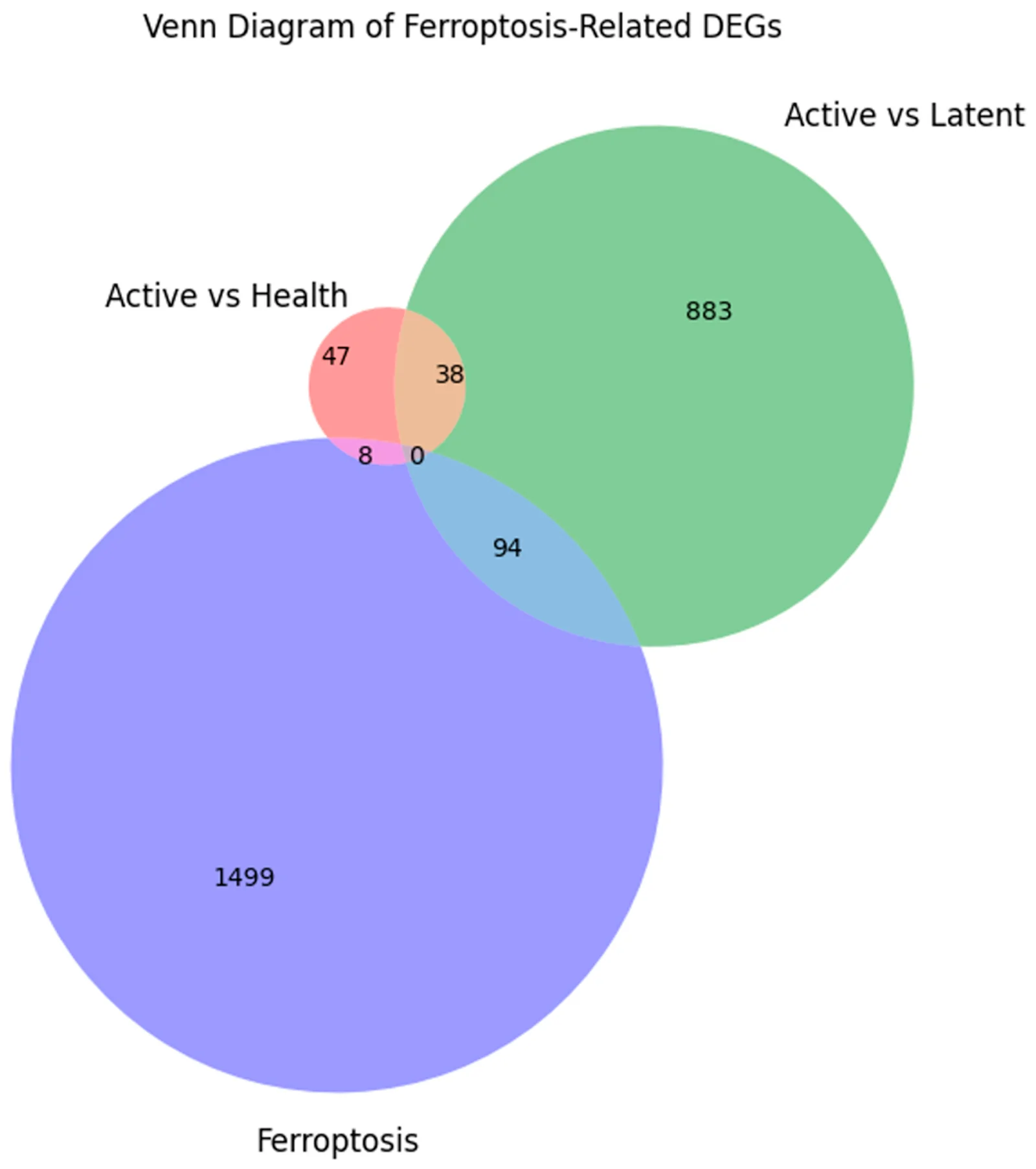
Intersection of TB differentially expressed genes with a ferroptosis-associated gene set in GSE37250. Venn diagram showing the overlap among DEGs identified from active TB vs. latent TB, DEGs identified from active TB vs. healthy controls, and ferroptosis-related genes. The analysis identified 94 ferroptosis-related DEGs in the active TB vs. latent TB comparison and 8 ferroptosis-related DEGs in the active TB vs. healthy comparison. A total of 38 genes were shared between the two DEG comparisons but did not overlap with the ferroptosis gene set. No genes were common to all three sets, suggesting comparison-specific ferroptosis-related transcriptional changes in active TB.

The intersection analysis identified 94 differentially expressed genes (DEGs) common to both the active tuberculosis (TB) versus latent TB comparison and the FerrDb-derived ferroptosis-associated gene set. Conversely, eight DEGs from the active tuberculosis versus healthy control comparison intersected with the ferroptosis-associated gene set. The two DEG comparisons shared 38 genes; however, none of these overlapped with the FerrDb-derived gene set, resulting in no genes being shared across all three sets under the applied filtering criteria. A re-examination of the source DEG lists and the FerrDb-derived gene set confirmed this outcome. The absence of a three-way overlap likely reflects the contrast-dependent nature of differential expression analysis, as the expression differences observed for active TB are contingent upon the comparator group and its underlying expression profile and variability. Therefore, the lack of common genes across all three sets should be interpreted as a comparison-specific finding rather than evidence of entirely distinct biological mechanisms. The 94-gene overlap represents a subset of transcriptional alterations associated with active TB that intersect with genes curated in FerrDb in relation to ferroptosis. Notably, this transcriptomic overlap does not establish ferroptotic activity or a causal role for ferroptosis in the progression from latent to active TB. These 94 intersection genes were subsequently utilized for functional enrichment, protein–protein interaction (PPI) network construction, and hub-gene prioritization (Figure 2). The complete list of the 94 intersecting genes and their FerrDb classifications is provided in Supplementary Table S1.

### 2.3. Functional Enrichment Profile of the 94 Ferroptosis-Associated Intersection Genes

**Figure 3:**
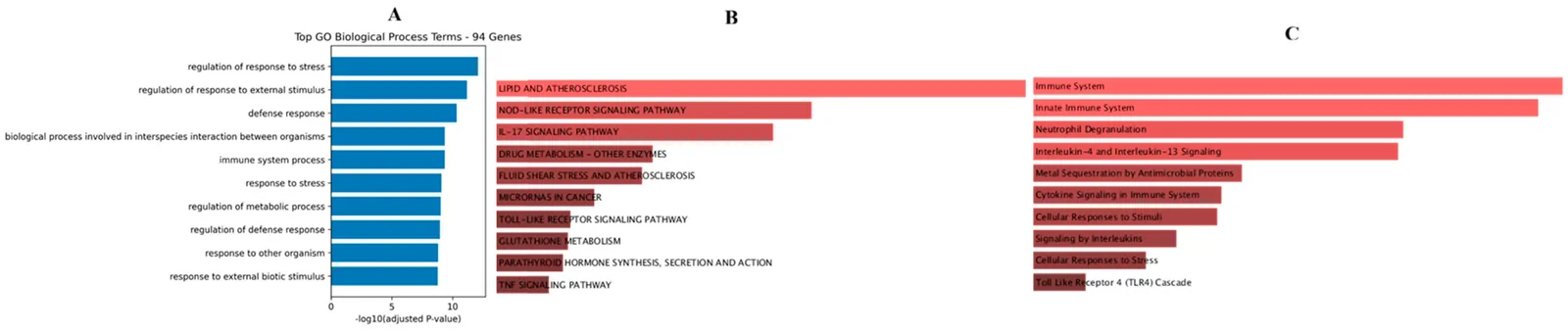
Functional enrichment of the 94 ferroptosis-associated intersection genes is depicted through bar plots, which rank the top enriched functional terms by −log10 adjusted *p*-value. Blue bars represent GO biological process terms, whereas red/pink bars represent KEGG and Reactome pathway terms; variations in color intensity are used for visual distinction only and do not encode an additional variable. (**A**) The Gene Ontology (GO) biological process enrichment analysis indicates that these DEGs are predominantly linked to the regulation of stress response, regulation of response to external stimuli, defense response, immune system processes, response to stress, regulation of defense response, response to other organisms, and response to external biotic stimuli. (**B**) The KEGG pathway enrichment analysis identifies significant associations with lipids and atherosclerosis, the NOD-like receptor signaling pathway, the IL-17 signaling pathway, the Toll-like receptor signaling pathway, glutathione metabolism, and the TNF signaling pathway. (**C**) The Reactome pathway enrichment analysis reveals involvement in the Immune System, Innate Immune System, Neutrophil Degranulation, Interleukin signaling, Metal Sequestration by Antimicrobial Proteins, Cytokine Signaling in the Immune System, Cellular Responses to Stimuli, and Toll-like receptor-related pathways. These results imply that the ferroptosis-related DEGs in active tuberculosis are primarily associated with immune activation, inflammatory signaling, neutrophil-mediated antimicrobial responses, oxidative/stress-related processes, and metabolic regulation linked to ferroptosis.

Functional enrichment analysis was performed on the 94 DEGs identified at the intersection between the active disease versus latent TB differential-expression set and the FerrDb-derived ferroptosis-associated gene set. GO biological process analysis revealed that these genes were predominantly enriched in immune- and stress-related processes, including regulation of response to stress, regulation of response to external stimulus, defense response, immune system process, response to stress, regulation of defense response, response to other organisms, and response to external biotic stimulus (Figure 3A). These findings suggest that the ferroptosis-related DEGs are strongly associated with host immune activation and stress-response mechanisms during active TB. Pathway enrichment analysis further supported the involvement of immune–inflammatory and ferroptosis-associated mechanisms. KEGG pathway analysis highlighted enrichment in Lipid and atherosclerosis, the NOD-like receptor signaling pathway, the IL-17 signaling pathway, the Toll-like receptor signaling pathway, glutathione metabolism, and the TNF signaling pathway (Figure 3B). Reactome pathway analysis showed enrichment in Immune System, Innate Immune System, Neutrophil Degranulation, Interleukin-4 and Interleukin-13 signaling, Metal Sequestration by Antimicrobial Proteins, Cytokine Signaling in Immune System, Cellular Responses to Stimuli, Signaling by Interleukins, and Toll-like receptor-related pathways (Figure 3C). Together, these results indicate that the 94 ferroptosis-related DEGs are mainly involved in immune activation, inflammatory signaling, neutrophil-mediated antimicrobial responses, oxidative/stress-related processes, and ferroptosis-associated metabolic regulation in active TB. GO molecular function enrichment indicated significant involvement in identical protein binding, enzyme binding, catalytic activity, transcription regulator activity, molecular adaptor activity, protein binding, transferase activity, protein dimerization activity, and transcription coregulator activity (Supplementary Figure S1A). GO cellular component enrichment demonstrated that the 94 ferroptosis-related DEGs were mainly localized to the cytosol, cytoplasm, intracellular organelle lumen, membrane-enclosed lumen, organelle lumen, vesicles, intracellular membrane-bounded organelles, mitochondria, and extracellular space (Supplementary Figure S1B). The complete g:Profiler enrichment output and gene–term intersection matrix for the 94 ferroptosis-related DEGs are provided in Supplementary Figure S3 and Supplementary Table S2.

### 2.4. PPI Network of the 94 Intersection Genes

**Figure 4:**
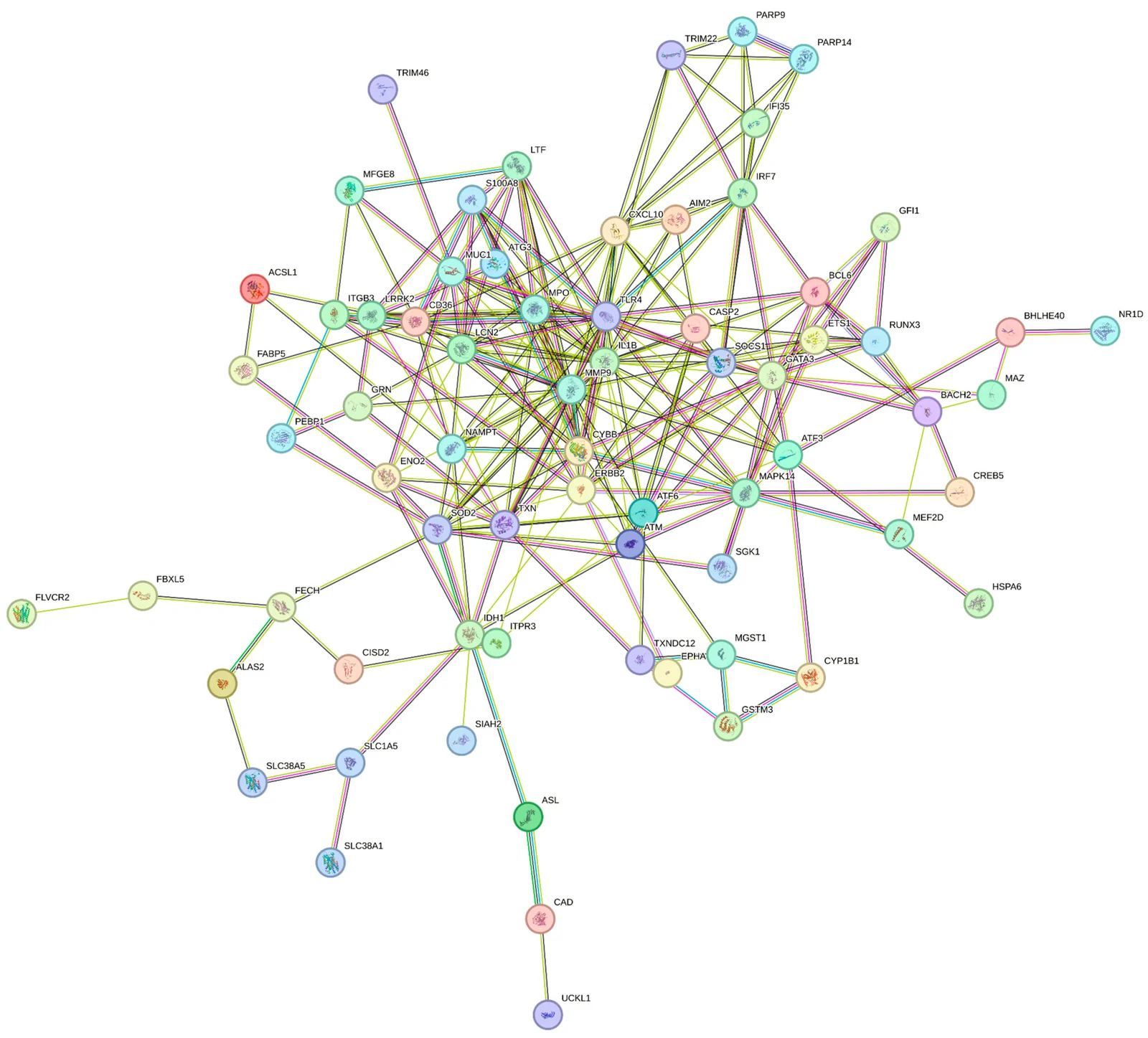
PPI network of the 94 ferroptosis-associated intersection genes. The protein–protein interaction network was constructed from 94 ferroptosis-related genes exhibiting differential expression between active and latent tuberculosis. This network, generated using STRING, illustrates functional associations among candidate proteins. In the STRING evidence view, edge colors indicate the type of supporting evidence: red, gene fusion; green, gene neighborhood; blue, gene co-occurrence; purple, experimental evidence; yellow, text mining; light blue, curated database evidence; and black, co-expression. A densely connected central module was identified, comprising genes related to immune response, inflammation, and oxidative stress, including IL1B, TLR4, CXCL10, MMP9, CYBB, MPO, CD36, LCN2, S100A8, and LTF. Peripheral clusters contained genes linked to lipid metabolism, iron regulation, amino acid transport, and cellular stress responses. Further analysis of the network was conducted using Cytoscape/cytoHubba to identify hub genes.

To examine the interaction patterns among the 94 ferroptosis-related intersection genes, a protein–protein interaction (PPI) network was constructed using the STRING database. The analysis revealed a highly interconnected central region, indicating that several ferroptosis-related DEGs may operate collectively within shared biological pathways. These pathways are implicated in immune activation, inflammatory signaling, oxidative stress, and host defense responses during active tuberculosis. The central region of the network encompassed numerous genes associated with immune and inflammatory responses, such as IL1B, TLR4, CXCL10, MMP9, CYBB, MPO, CD36, LCN2, S100A8, and LTF. These genes exhibited close connections with other inflammatory and stress-related genes, including IRF7, IFI35, PARP9, PARP14, SOCS1, GATA3, ATF3, MAPK14, SOD2, TXN, NAMPT, and ERBB2. The presence of these interactions corroborates the involvement of ferroptosis-related DEGs in coordinated immune and oxidative stress responses. Beyond the central immune-related cluster, several peripheral interaction groups were identified. These groups included genes linked to lipid metabolism, iron handling, amino acid transport, and stress regulation, such as ACSL1, FABP5, ALAS2, FECH, FBXL5, FLVCR2, SLC1A5, SLC38A1, and SLC38A5 (Figure 4). This network structure implies that transcriptional changes related to ferroptosis in active tuberculosis may encompass both immune–inflammatory mechanisms and metabolic pathways pertinent to ferroptosis biology. The PPI network was processed using Cytoscape version 3.10.3. and the cytoHubba plugin to rank highly connected hub genes for subsequent prioritization and validation.

### 2.5. Functional Enrichment Profile of the 10 Hub Genes

**Figure 5:**
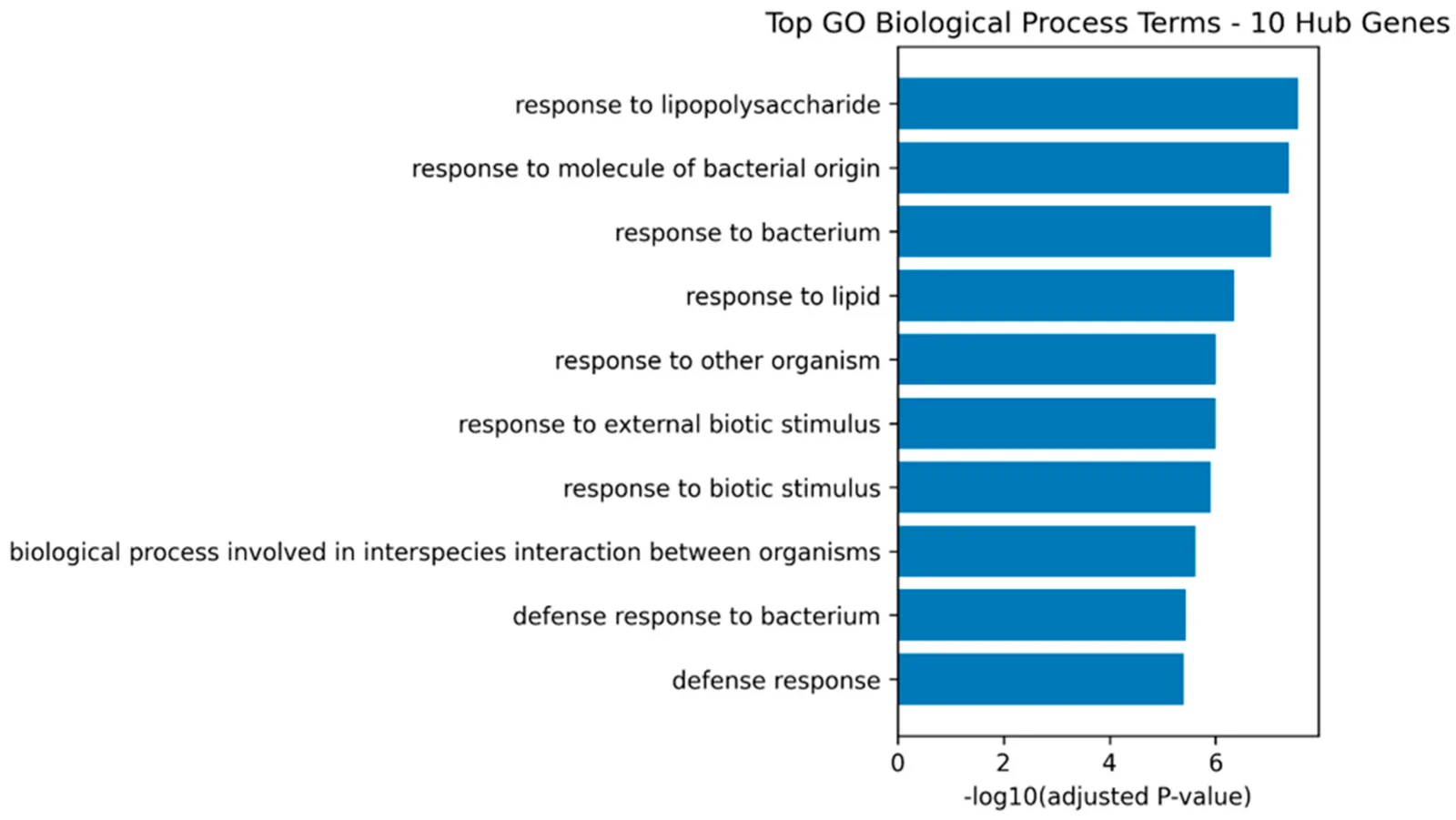
The enrichment analysis of Gene Ontology (GO) biological processes for the 10 hub genes is presented in a horizontal bar plot, which ranks the top enriched GO biological process terms by the −log10 adjusted *p*-value. The analysis reveals that the 10 hub genes are enriched in processes such as response to lipopolysaccharide, response to molecules of bacterial origin, response to bacterium, response to lipid, response to other organisms, response to external biotic stimuli, response to biotic stimuli, biological processes involved in interspecies interaction, defense response to bacterium, and defense response. These results suggest a strong association of the prioritized hub genes with bacterial recognition, innate immune activation, host defense, and inflammatory responses in active tuberculosis.

To evaluate the biological significance of the prioritized hub genes, a Gene Ontology (GO) enrichment analysis was performed on the 10 selected hub genes. The GO biological process enrichment analysis demonstrated a strong association with responses to lipopolysaccharide, molecules of bacterial origin, bacteria, lipids, other organisms, external biotic stimuli, biotic stimuli, and biological processes involved in interspecies interaction, defense response to bacteria, and general defense response (Figure 5). These findings indicate that the final hub genes are closely linked to bacterial recognition, host defense, and inflammatory immune responses. The GO molecular function assessment of the 10 selected hub genes revealed enrichment in Toll-like receptor binding, heparin binding, lipopolysaccharide binding, glycosaminoglycan binding, sulfur compound binding, pattern recognition receptor activity, and signaling receptor binding (Supplementary Figure S2A). The GO cellular component enrichment analysis indicated that these genes are primarily localized in secretory granules, secretory vesicles, phagocytic vesicles, specific granules, cytoplasmic vesicles, intracellular vesicles, phagocytic vesicle lumen, extracellular space, secretory granule lumen, and cytoplasmic vesicle lumen (Supplementary Figure S2B). These results support the involvement of the hub genes in innate immune recognition, bacterial response, secretory/phagocytic activity, neutrophil-related processes, and inflammatory host defense mechanisms in active tuberculosis. The complete g:Profiler enrichment output for the 10 hub genes is provided in Supplementary Figure S4 and Supplementary Table S3.

### 2.6. Identification of the 10 Hub Genes

**Figure 6:**
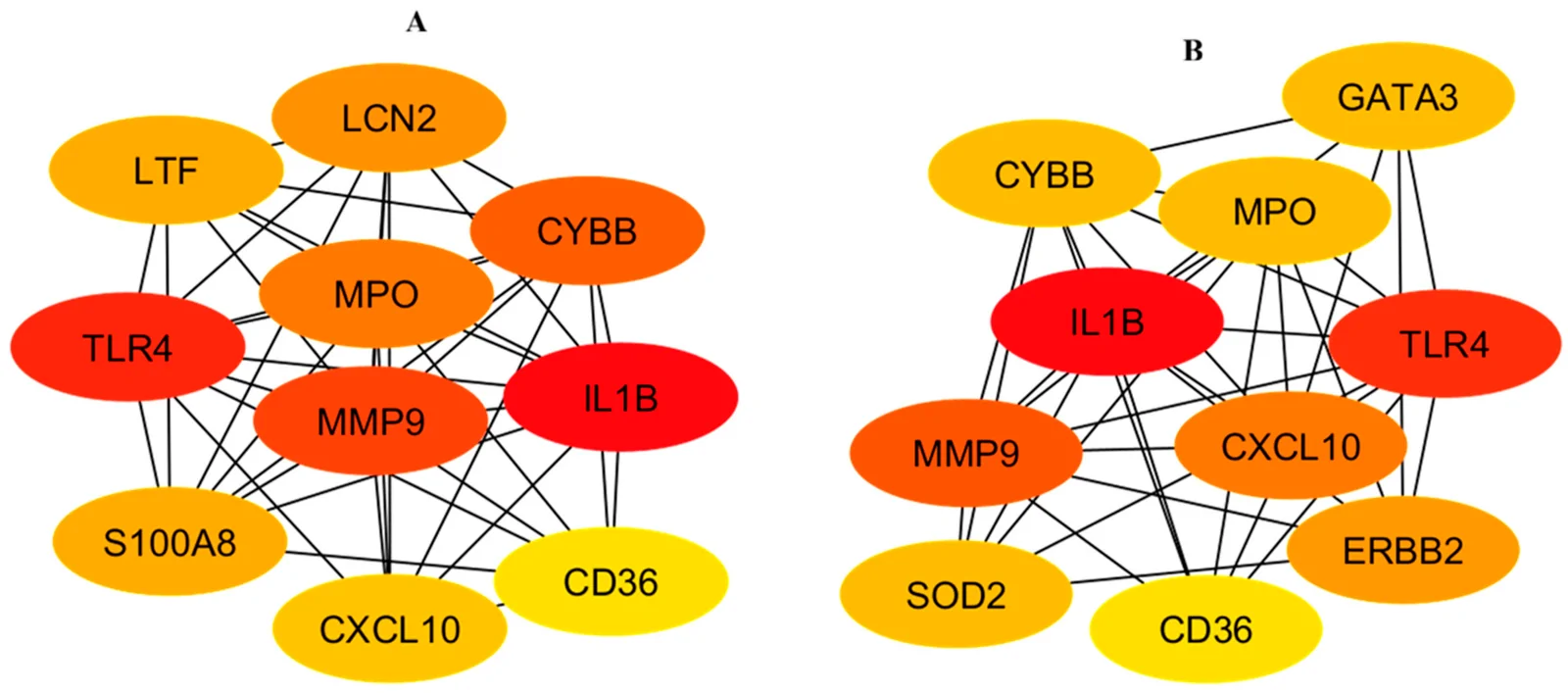
Hub-gene identification using cytoHubba. Hub genes were identified from the PPI network of the 94 ferroptosis-related DEGs using the cytoHubba plugin in Cytoscape. (**A**) Top 10 hub genes ranked by the MCC algorithm, including IL1B, TLR4, MMP9, MPO, CYBB, LCN2, LTF, S100A8, CXCL10, and CD36. (**B**) Top 10 hub genes ranked by the Degree algorithm, including IL1B, TLR4, CXCL10, MMP9, CYBB, MPO, CD36, ERBB2, SOD2, and GATA3. Seven genes overlapped between the two algorithms: IL1B, TLR4, CXCL10, MMP9, CYBB, MPO, and CD36. These overlapping genes were considered robust core hubs, while LCN2, S100A8, and LTF were additionally retained from the MCC ranking due to their biological relevance to neutrophil-associated antimicrobial and ferroptosis-related responses.

To identify the most influential genes within the PPI network, the 94 ferroptosis-related DEGs were further analyzed using the cytoHubba plugin in Cytoscape. Two topological ranking algorithms, MCC and Degree, were employed to enhance the robustness of hub-gene selection. The MCC algorithm identified the top 10 hub genes as IL1B, TLR4, MMP9, MPO, CYBB, LCN2, LTF, S100A8, CXCL10, and CD36 (Figure 6A). These genes are primarily associated with inflammatory signaling, innate immune activation, neutrophil-related antimicrobial responses, oxidative stress, lipid metabolism, and ferroptosis-related host defense mechanisms. The Degree algorithm identified the top 10 hub genes as IL1B, TLR4, CXCL10, MMP9, CYBB, MPO, CD36, ERBB2, SOD2, and GATA3 (Figure 6B). This ranking similarly emphasized genes involved in inflammatory response, Toll-like receptor signaling, chemokine-mediated immune activation, oxidative stress, and cellular defense pathways. A comparison of the MCC and Degree results revealed seven overlapping hub genes: IL1B, TLR4, CXCL10, MMP9, CYBB, MPO, and CD36. These overlapping genes were considered the most robust core hub genes, as they were prioritized by both algorithms. Additionally, LCN2, S100A8, and LTF were retained from the MCC top-ranked genes due to their biological relevance to neutrophil activation, antimicrobial responses, and metal sequestration pathways. Consequently, a final panel of 10 hub genes, comprising IL1B, TLR4, CXCL10, MMP9, CYBB, MPO, CD36, LCN2, S100A8, and LTF, was selected for downstream validation and functional interpretation.

### 2.7. External Validation Results of the Hub Genes

**Figure 7:**
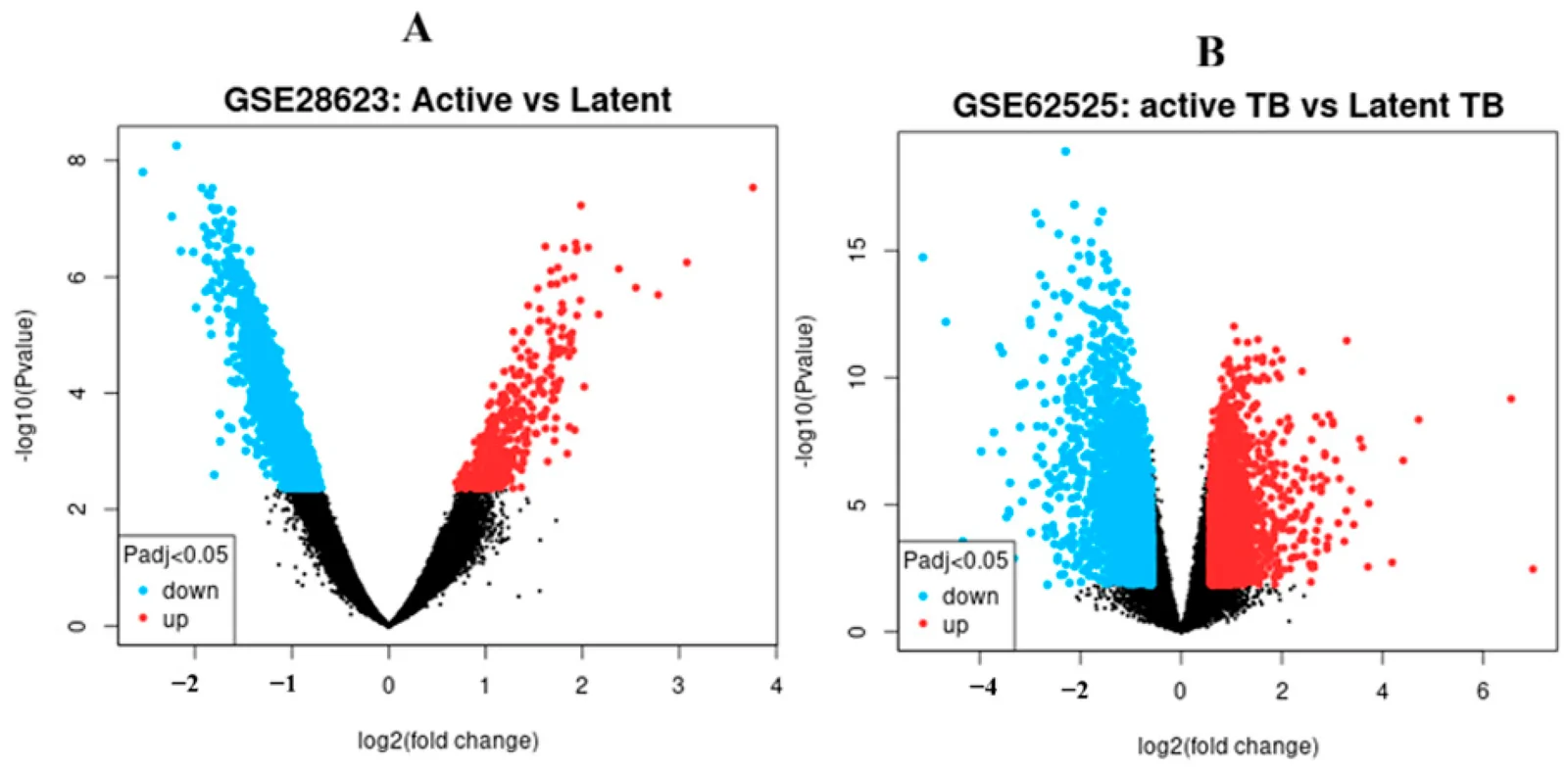
Differential expression analysis in external validation datasets. Volcano plots showing differential gene expression between active TB and latent TB in two independent validation datasets. (**A**) GSE28623 active TB versus latent TB. (**B**) GSE62525 active TB versus latent TB. The x-axis represents log2 fold change, and the y-axis represents −log10 *p*-value. Red dots indicate significantly upregulated genes, blue dots indicate significantly downregulated genes, and black dots indicate nonsignificant genes based on adjusted *p*-value < 0.05. These datasets were used to evaluate the expression direction and statistical significance of the final 10 hub genes.

Following the selection of probes independent of outcomes, external validation revealed variable reproducibility between the two datasets (Figure 7). LCN2 and LTF exhibited the strongest cross-dataset replication, with significant positive differential expression observed in both GSE28623 and GSE62525. IL1B, TLR4, MMP9, and MPO were significantly upregulated in GSE62525 and displayed concordant positive, albeit nonsignificant, logFC values in GSE28623. It is noteworthy that MMP9 maintained a strong positive expression in GSE28623 but did not achieve the adjusted *p*-value threshold after revised probe selection (logFC = 0.933, adjusted *p* = 0.0544). CXCL10, CYBB, and CD36 exhibited positive but nonsignificant logFC values in both validation datasets, thus lacking independent validation. S100A8 was significantly upregulated in GSE28623 but demonstrated a small negative and nonsignificant logFC in GSE62525, suggesting dataset-dependent expression. In summary, LCN2 and LTF emerge as the strongest candidates across the two independent validation datasets (Table 1).

When multiple probes mapped to the same gene symbol, the representative probe was selected based on the highest mean normalized expression intensity across all samples included in the active TB group versus LTBI comparison, independently of differential-expression statistics. A gene was considered statistically validated when it showed a positive logFC and an adjusted *p*-value < 0.05. Positive logFC with an adjusted *p*-value ≥ 0.05 was considered directionally concordant but not statistically validated, whereas an opposite expression direction was considered directionally inconsistent.

**Figure 8:**
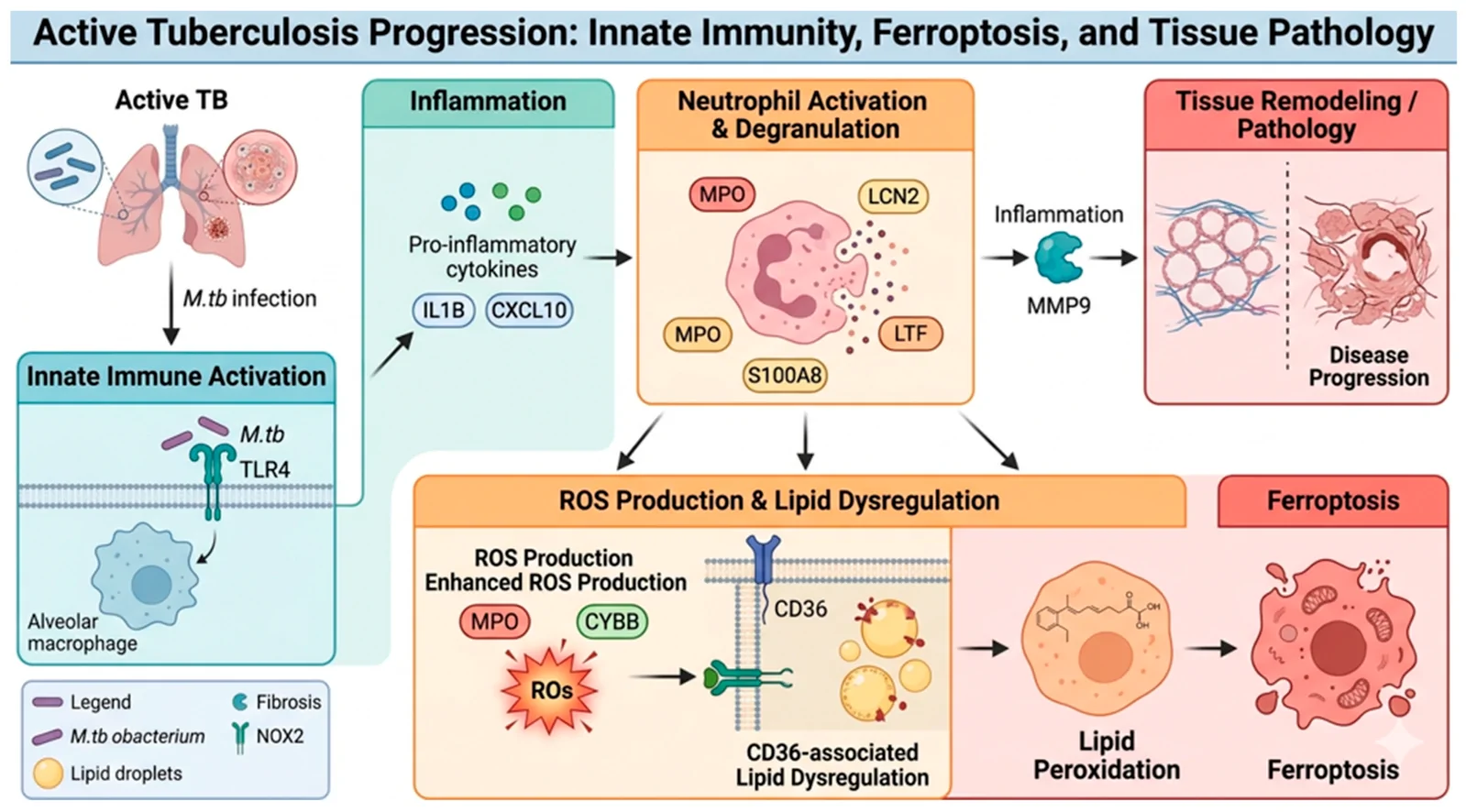
This framework proposes a link between the immune–inflammatory transcriptional network and ferroptosis-related biological processes in active tuberculosis. The figure is a conceptual illustration and does not show experimental data or a proven causal mechanism. Source: Created using ChatGPT (OpenAI) following the authors’ instructions; the authors checked and confirmed the scientific content of the final figure.

A hypothesis-generating framework was developed to summarize the potential biological relationships among the identified hub genes (Figure 8). This model integrates observed transcriptomic and network findings with biological processes such as innate immune activation, inflammatory signaling, neutrophil degranulation, ROS generation, lipid metabolism, and tissue remodeling. Although several of these processes have established biological links with ferroptosis, the current transcriptomic analysis does not demonstrate lipid peroxidation, iron-dependent cell death, or ferroptosis itself. Therefore, the relationship between the identified network and ferroptosis-associated processes is presented as a hypothesis that requires experimental validation.

## 3. Discussion

Tuberculosis continues to pose a significant global health challenge, with the differentiation between active tuberculosis (TB) and latent tuberculosis infection being clinically crucial. This distinction is important because active TB represents a transmissible infection and involves progressive host–pathogen interactions. In this study, an integrated transcriptomic and systems-biology approach was employed to identify ferroptosis-related genes associated with the progression of active TB. The analysis demonstrated that the transcriptional dysregulation observed in the comparison between active TB and latent TB was more extensive than that between active TB and healthy controls, thereby justifying the use of the active TB versus latent TB comparison as the primary discovery contrast. This finding is biologically significant, as the transition from latent infection to active disease is a pivotal stage in TB pathogenesis, characterized by immune dysregulation, inflammatory activation, metabolic remodeling, and tissue injury. A key finding of this study was the identification of 94 ferroptosis-related differentially expressed genes (DEGs) in the active disease state versus latent disease state comparison. This indicates that ferroptosis-related transcriptional dysregulation is more closely associated with the transition from latent infection to active disease than with the general comparison between active TB and healthy controls. Ferroptosis is an iron-dependent form of regulated cell death driven by lipid peroxidation and oxidative stress, regulated by cellular pathways involved in iron metabolism, redox balance, mitochondrial activity, amino acid metabolism, and lipid metabolism [10,11]. These biological characteristics are highly pertinent to TB, as *Mycobacterium tuberculosis* infection is known to alter host iron handling, induce oxidative stress, and promote inflammatory tissue injury. Experimental evidence has demonstrated that *M. tuberculosis*-induced macrophage necrosis can involve ferroptosis-associated mechanisms, including iron accumulation, lipid peroxidation, and suppression of GPX4-dependent antioxidant protection [6]. The enrichment results further corroborate the biological relevance of the identified ferroptosis-related DEGs. Gene Ontology (GO) biological process enrichment highlighted immune response, defense response, response to stress, and response to external biotic stimulus, indicating that the ferroptosis-related gene set is closely linked to host immune activation during active TB. KEGG and Reactome enrichment analyses also revealed pathways related to Toll-like receptor signaling, NOD-like receptor signaling, IL-17 signaling, TNF signaling, glutathione metabolism, neutrophil degranulation, interleukin signaling, and metal sequestration by antimicrobial proteins. These findings are consistent with previous transcriptomic studies showing that active TB is characterized by strong inflammatory and innate immune transcriptional signatures. Berry et al. (2010) described an interferon-inducible, neutrophil-driven blood transcriptional signature in active TB, while Ottenhoff et al. (2012) identified type I interferon response pathways as prominent features of active TB [2,4]. Kaforou, also demonstrated that whole-blood RNA signatures can distinguish active TB from latent TB and other disease states, supporting the diagnostic and biological value of blood transcriptomics in TB research [3]. The PPI network and cytoHubba analyses identified a final panel of 10 prioritized hub genes: IL1B, TLR4, CXCL10, MMP9, CYBB, MPO, CD36, LCN2, S100A8, and LTF. Notably, seven of these genes—IL1B, TLR4, CXCL10, MMP9, CYBB, MPO, and CD36—were common to both the MCC and Degree algorithms, indicating significant network centrality. Additionally, LCN2, S100A8, and LTF were retained from the MCC ranking due to their biological relevance to neutrophil activation, antimicrobial defense, and metal sequestration. This dual prioritization strategy enhances the study by avoiding reliance on a single network-ranking method and integrating topological robustness with biological plausibility. The hub-gene profile suggests that ferroptosis-related dysregulation in active TB is intricately linked to innate immune recognition and inflammatory signaling. TLR4, a pattern-recognition receptor, plays a crucial role in detecting pathogen-associated and damage-associated molecular patterns and activating downstream inflammatory pathways. Previous studies have underscored the significance of Toll-like receptor signaling in the innate immune response to *M. tuberculosis*, with a systematic review specifically highlighting TLR4 as an immune receptor involved in *M. tuberculosis* infection outcomes [12]. The identification of TLR4 as a hub gene in this study aligns with recent ferroptosis-related TB bioinformatics studies that identified TLR4 among candidate ferroptosis-associated biomarkers for active TB [9,13]. The inclusion of IL1B and CXCL10 among the prioritized hub genes further corroborates the inflammatory nature of the ferroptosis-related TB signature. IL1B is a pivotal pro-inflammatory cytokine involved in innate immune activation, while CXCL10/IP-10 is an interferon-inducible chemokine extensively investigated as a biomarker of TB infection and active disease. Previous research has demonstrated that CXCL10/IP-10 can differentiate active TB from latent TB and may decrease during treatment, indicating its reflection of active inflammatory burden [14]. In the context of the present study, the inclusion of CXCL10 supports the association between interferon-associated immune activation and ferroptosis-related transcriptional changes. Despite CXCL10 exhibiting positive differential expression in both external datasets, it failed to achieve statistical significance in either dataset and thus was not independently validated. Another significant finding was the prioritization and external validation of neutrophil-associated genes, particularly MMP9, LCN2, LTF, MPO, and S100A8. Neutrophils have been strongly implicated in active TB transcriptional signatures and TB immunopathology. Berry et al. (2010) reported that patients with active TB exhibited a whole-blood transcriptional signature dominated by a neutrophil-driven interferon-inducible gene profile. while subsequent studies demonstrated that neutrophil-associated proteins such as S100A8/A9 contribute to neutrophilic inflammation and lung pathology during TB [2]. The enrichment of secretory granules, phagocytic vesicles, neutrophil degranulation, and metal sequestration pathways in this study provides robust biological support for the hub-gene panel.

The findings indicate variable cross-dataset reproducibility among the prioritized hub genes. LCN2 and LTF emerge as the most strongly supported candidates, having satisfied the statistical validation criteria in both independent datasets. IL1B, TLR4, and CD36 received partial support, while CYBB and MPO exhibited directional concordance without achieving statistical significance. The inconsistent expression of CXCL10 and S100A8 across datasets suggests that the entire 10-gene panel should not be regarded as a universally reproducible active TB signature. MMP9 is particularly pertinent to tuberculosis (TB) due to the role of matrix metalloproteinases in extracellular matrix degradation, lung tissue destruction, and TB-associated immunopathology. Elkington et al. highlighted that MMPs may play a central role in TB pathology by degrading lung extracellular matrix components. MMP9 remains biologically significant in tuberculosis (TB) due to its role in matrix metalloproteinases, which contribute to extracellular matrix degradation, lung tissue destruction, and TB-associated pathology. In the current external validation analysis, MMP9 was notably upregulated in GSE62525 and exhibited a consistent positive differential expression in GSE28623. However, it narrowly missed the adjusted *p*-value threshold in the latter dataset (adjusted *p* = 0.0544). Consequently, the external evidence for MMP9 should be regarded as supportive, though not statistically replicated across both datasets. These findings support further investigation of MMP9 as a candidate linking inflammatory responses with tissue-remodeling processes in active TB.

LCN2 and LTF exhibited significant positive differential expression in both validation datasets, representing the most robust cross-dataset evidence among the prioritized hub genes. These proteins are integral to innate antimicrobial defense and nutritional immunity. LCN2 enhances host defense by binding siderophores and limiting microbial iron acquisition, while LTF, an iron-binding antimicrobial glycoprotein, is prevalent in neutrophil granules and mucosal secretions. The consistent expression of these genes across independent TB datasets warrants further exploration of iron-related nutritional immunity, neutrophil-associated responses, and antimicrobial host defense mechanisms in active TB. Notably, although both genes are part of the FerrDb-derived ferroptosis-associated gene set, their transcriptional upregulation in this study does not confirm a direct involvement in ferroptotic cell death [15].

The identification of CD36 further broadens the biological interpretation of this study by linking the hub-gene panel to lipid uptake and lipid dysregulation. CD36, a scavenger receptor involved in lipid uptake, has been shown in previous research to facilitate the uptake of surfactant lipids by human macrophages, promoting the intracellular growth of *M. tuberculosis* [16]. This is relevant to ferroptosis, as it is driven by lipid peroxidation, and lipid remodeling can influence susceptibility to ferroptosis. Moreover, *M. tuberculosis* is known to induce lipid-laden foamy macrophage phenotypes, which support bacterial persistence and contribute to TB pathology. Thus, the inclusion of CD36 in the final hub-gene panel provides a biologically plausible connection between TB-associated lipid metabolism and ferroptosis-related injury [16]. CD36 exhibited positive, albeit nonsignificant, differential expression in both validation datasets. Consequently, it should be considered a biologically plausible network candidate rather than an externally validated marker.

The oxidative stress-related genes CYBB and MPO also support the proposed model. CYBB encodes a component of the NADPH oxidase complex involved in reactive oxygen species generation, while MPO is a neutrophil granule enzyme involved in oxidative antimicrobial activity. The oxidative-stress-associated genes CYBB and MPO exhibited varying levels of external validation. In the GSE62525 dataset, MPO was significantly upregulated, while for GSE28623, it demonstrated a positive yet nonsignificant expression. Conversely, CYBB displayed positive but nonsignificant differential expression in both datasets. These results partially support the genes’ involvement in the identified immune–inflammatory network, although they do not confirm their role in ferroptosis. CYBB and MPO are biologically pertinent to the generation of reactive oxygen species and neutrophil antimicrobial responses, which may intersect with oxidative-stress pathways linked to ferroptosis. However, this study did not directly evaluate lipid peroxidation and ferroptotic cell death.

The results of external validation indicated variable cross-dataset reproducibility among the prioritized hub genes. LCN2 and LTF exhibited the most robust evidence of replication, as both genes showed significant positive differential expression in the GSE28623 and GSE62525 datasets. IL1B, TLR4, MMP9, and MPO were significantly upregulated in GSE62525 but displayed positive, nonsignificant differential expression in GSE28623. Notably, MMP9 remained strongly positive in GSE28623 but narrowly failed to meet the adjusted *p*-value threshold following outcome-independent probe selection (logFC = 0.933, adjusted *p* = 0.0544). CXCL10, CYBB, and CD36 demonstrated positive but nonsignificant differential expression in both validation datasets, whereas S100A8 was significantly upregulated in GSE28623 but exhibited a small negative and nonsignificant logFC in GSE62525. Consequently, the entire 10-gene panel should not be regarded as a universally reproducible active TB signature, with LCN2 and LTF emerging as the most consistently supported candidates across the two independent datasets. This pattern warrants careful interpretation: the study does not establish that all 10 genes are universal biomarkers, but it provides compelling evidence that a subset of ferroptosis-related immune–inflammatory genes is consistently associated with active TB. Compared with previous studies on ferroptosis-related TB, which identified a ferroptosis-related gene signature comprising ACSL4, CTSB, and TLR4 [8], reported diagnostic potential across multiple blood datasets [10]. And identified ACSL1, PARP9, TLR4, and ATG3 as immune–inflammatory hub genes, the present study contributes by integrating multiple analytical layers into a cohesive framework and identifying ferroptosis-related DEGs that distinguish active TB from latent TB. The present study differs by identifying a broader immune–neutrophil–ferroptosis network and prioritizing genes such as MMP9, LCN2, LTF, S100A8, MPO, and CD36, which connect ferroptosis biology to neutrophil degranulation, antimicrobial metal sequestration, oxidative stress, lipid dysregulation, and tissue remodeling. This methodology builds upon prior research by incorporating immune–inflammatory, neutrophil-associated, oxidative, metabolic, and tissue-remodeling processes into a transcriptional network that intersects with genes associated with ferroptosis. A key strength of the study is the clear biological consistency across independent analytical steps. The DEG analysis identified a strong transcriptional shift in active TB; the ferroptosis intersection selected a focused gene set; enrichment analysis highlighted immune response, stress response, glutathione metabolism, lipid-related pathways, neutrophil degranulation, and Toll-like receptor signaling; PPI analysis demonstrated network connectivity; cytoHubba prioritized immune–inflammatory and neutrophil-associated hub genes; and external validation supported several central genes. This stepwise consistency enhances the reliability of the findings and supports the proposed mechanism model. The proposed mechanism model outlines how *M. tuberculosis* infection may induce innate immune activation through TLR4, followed by inflammatory signaling involving IL1B and CXCL10. Neutrophil-associated genes, including MPO, LCN2, LTF, and S100A8, may contribute to neutrophil activation, degranulation, antimicrobial metal sequestration, and inflammatory tissue injury. CYBB and MPO potentially play roles in processes related to oxidative stress, while CD36 may connect the network to lipid uptake and remodeling. Given the biological significance of oxidative stress and lipid metabolism in ferroptosis, these findings indicate a possible interaction with ferroptosis-associated biology. Nevertheless, this study did not directly evaluate lipid peroxidation or ferroptotic cell death.

MMP9 may further contribute to tissue remodeling and pathology. This model aligns with the known biology of ferroptosis as an iron- and lipid-peroxidation-dependent process and with experimental evidence linking ferroptosis to *M. tuberculosis*-induced necrosis and tissue injury. Despite the strengths of this study, an essential limitation of this study is the lack of direct assessment of ferroptosis. While the current analysis highlights transcriptional alterations that overlap with a curated set of genes associated with ferroptosis, this overlap does not confirm the occurrence of ferroptotic cell death. The public datasets analyzed do not include measurements of lipid reactive oxygen species (ROS) or lipid peroxidation, intracellular labile iron, GPX4 protein expression or activity, glutathione levels, lipid-peroxidation products such as malondialdehyde, pharmacological intervention with ferroptosis inhibitors, or ultrastructural changes. As a result, the connection between the identified transcriptional network and ferroptosis remains speculative. Experimental validation will require the use of suitable cellular or animal models of *M. tuberculosis* infection that incorporate biochemical, pharmacological, and morphological criteria of ferroptosis.

Overall, this study offers an integrated transcriptomic framework that links ferroptosis-related gene dysregulation with immune activation, neutrophil-mediated inflammation, oxidative stress, lipid remodeling, metal sequestration, and tissue pathology in active TB. This study presents a comprehensive transcriptomic framework that identifies immune–inflammatory genes associated with active tuberculosis (TB) and their intersection with a ferroptosis-associated gene set derived from FerrDb. Among the prioritized hub genes, LCN2 and LTF exhibited the highest cross-dataset reproducibility, while the other genes displayed partial, nonsignificant, or dataset-dependent external evidence. The broader network underscores biological processes such as innate immune activation, neutrophil responses, oxidative stress, lipid metabolism, metal sequestration, and tissue remodeling. Notably, the current findings do not establish ferroptosis or ferroptosis-mediated immunopathology. Instead, they offer a hypothesis-generating framework and candidate genes for future experimental exploration of the potential relationship between TB-associated host responses and ferroptosis-associated biology.

## 4. Materials and Methods

### 4.1. Microarray Datasets and GEO Database

Publicly accessible human tuberculosis transcriptomic datasets were retrieved from NCBI’s Gene Expression Omnibus (GEO), an open access resource that hosts gene-expression profiles and other functional genomics data generated by high-throughput studies, encompassing both microarray and sequencing-based expression profiles. The discovery dataset employed in this study is GSE37250, titled “Genome-wide transcriptional profiling of HIV-positive and HIV-negative adults with active tuberculosis, latent TB infection, and other diseases.” This dataset was produced using the GPL10558 Illumina HumanHT-12 V4.0 Expression BeadChip platform (Illumina, Inc., San Diego, CA, USA) and comprises whole-blood samples collected in PAX gene tubes. For the current analysis, samples categorized as active tuberculosis (active TB), latent tuberculosis infection (latent TB), and healthy controls were selected. Two independent GEO datasets, GSE28623 and GSE62525, were utilized for external validation. GSE28623 contains whole-blood gene expression profiles derived from TB-related clinical groups using human whole-genome expression arrays. According to GEO, whole-blood RNA was extracted using the PAXgene Blood RNA Kit (PreAnalytiX GmbH, Hombrechtikon, Switzerland; produced by QIAGEN GmbH, Hilden, Germany) and hybridized to whole-genome human expression arrays. GSE62525 served as an additional validation dataset and includes peripheral blood mononuclear cell expression profiles comparing active TB, latent TB infection, and healthy conditions within a Taiwanese population. The study’s overall workflow encompassed differential expression analysis, identification of ferroptosis-related differentially expressed genes, functional enrichment analysis, protein–protein interaction network construction, hub-gene identification, external validation, and the development of an integrative mechanism model.

### 4.2. Differential Expression Analysis

Differential expression analysis was conducted using GEO2R, an online tool provided by NCBI GEO (https://www.ncbi.nlm.nih.gov/geo/geo2r/; accessed on 8 March 2026) for comparing gene expression across sample groups. GEO2R employs the R packages GEOquery and limma to analyze submitter-supplied processed microarray data tables, facilitating the identification of differentially expressed genes between predefined groups. The limma package, which applies linear modeling and empirical Bayes moderation, is extensively utilized for differential expression analysis of microarray and RNA-seq data [17]. In the discovery dataset, GSE37250, two comparisons were executed: active TB vs. latent TB and active TB vs. healthy controls. Differentially expressed genes were identified using an adjusted *p*-value threshold of <0.05. Genes exhibiting positive log2 fold-change values were classified as upregulated, while those with negative log2 fold-change values were classified as downregulated. Volcano plots were generated to depict the distribution of differentially expressed genes in each comparison. Red points indicated significantly upregulated genes, blue points indicated significantly downregulated genes, and black points indicated nonsignificant genes. The comparison between active TB and latent TB was selected as the principal discovery comparison due to its demonstration of the largest transcriptional shift and the highest number of significant DEGs.

### 4.3. Identification of Differentially Expressed Genes Intersecting with a Ferroptosis-Associated Gene Set

To explain transcriptional changes associated with tuberculosis (TB) that intersect with ferroptosis-related biology, significant differentially expressed genes (DEGs) from the GSE37250 dataset were compared with a curated set of ferroptosis-associated genes obtained from FerrDb V3 (https://www.zhounan.org/ferrdb/; accessed on 12 March 2026). FerrDb comprises genes categorized based on their documented associations with ferroptosis, including drivers, suppressors, markers, and genes with partially classified regulatory roles. In this study, genes common to both the active TB versus latent TB DEG list and the FerrDb-derived gene set were identified as DEGs intersecting with the ferroptosis-associated gene set. It is crucial to note that inclusion in this intersection indicates a transcriptomic association and does not imply direct regulation or the occurrence of ferroptosis in TB. The full list of the 94 intersecting genes and their FerrDb classifications is available in Supplementary Table S1.

A Venn diagram was generated to illustrate the overlap among the three gene sets. Genes common to both the active TB vs. latent TB DEG list and the ferroptosis-related gene set were defined as the main ferroptosis-related DEGs and were subsequently used for downstream functional enrichment and network analyses.

### 4.4. Functional Enrichment Analysis of the 94 Ferroptosis-Associated Intersection Genes

Functional enrichment analysis was conducted using g:Profiler/g:GOSt, focusing on Homo sapiens. The analysis covered Gene Ontology Biological Process (GO:BP), Gene Ontology Molecular Function (GO:MF), Gene Ontology Cellular Component (GO:CC), Kyoto Encyclopedia of Genes and Genomes (KEGG), Reactome, WikiPathways, Human Protein Atlas, and CORUM datasets. Terms with an adjusted *p*-value below 0.05 were deemed statistically significant. The g:Profiler analysis used version e114_eg62_p19_27110d83, accessed on 8 July 2026. Clean enrichment bar plots were created in Google Colaboratory using Python version 3.10.3 from the g:Profiler result tables. Terms were ranked by −log10 adjusted *p*-value. The main manuscript highlighted GO biological process, KEGG, and Reactome enrichment results, while GO molecular function, GO cellular component, and full g:Profiler outputs were included as Supplementary Figures.

### 4.5. Protein–Protein Interaction Network Construction of the 94 Intersection Genes

To explore potential functional interactions among the 94 ferroptosis-related DEGs, a protein–protein interaction (PPI) network was constructed using the STRING database v12.0. STRING integrates known and predicted protein–protein associations derived from experimental data, computational predictions, co-expression, curated databases, and text mining. The STRING database is widely utilized for constructing functional association networks and conducting enrichment analysis. The 94 ferroptosis-related DEGs were uploaded to STRING with Homo sapiens selected as the organism, and a minimum interaction confidence score of 0.4 was applied. The resulting PPI network was visualized using Cytoscape software version 3.10.3., an open-source platform for visualizing and analyzing molecular interaction networks. Cytoscape version 3.10.3. is commonly employed for biological network visualization, annotation, and topological analysis [18].

### 4.6. Hub-Gene Identification Using cytoHubba

Hub genes were identified from the PPI network using the cytoHubba plugin in Cytoscape. cytoHubba is a Cytoscape plugin designed to identify significant nodes and hub objects in biological networks based on topological ranking algorithms [19,20]. Two ranking algorithms were employed: Maximal Clique Centrality (MCC) and Degree. The top 10 genes ranked by the MCC algorithm and the top 10 genes ranked by the Degree algorithm were extracted. The overlap between the two ranking methods was then determined to identify robust core hub genes. Genes present in both the MCC and Degree top-10 lists were considered overlapping hub genes. Additionally, biologically relevant genes present in the MCC top-10 list were retained when strongly associated with neutrophil activation, antimicrobial responses, or ferroptosis-related immune pathways. The final prioritized hub-gene panel included IL1B, TLR4, CXCL10, MMP9, CYBB, MPO, CD36, LCN2, S100A8, and LTF. These genes were used for downstream enrichment interpretation, external validation, and mechanism-model construction.

### 4.7. Functional Enrichment Analysis of the Final Hub Genes

To further elucidate the biological significance of the final hub-gene panel, a functional enrichment analysis was conducted on the 10 selected hub genes using g:Profiler/g:GOSt. The main manuscript employed GO biological process enrichment to encapsulate the principal biological functions associated with these hub genes. Supplementary Materials included enrichment plots for GO molecular function and GO cellular component. The same significance threshold was maintained, with enriched terms deemed significant at an adjusted *p*-value < 0.05. Enriched terms were ranked by −log10 adjusted *p*-value and visualized as horizontal bar plots.

### 4.8. External Validation of Hub Genes

External validation was conducted using the GSE28623 and GSE62525 datasets to compare active tuberculosis (TB) with latent TB. For each hub gene, the log fold-change (logFC), *p*-value, adjusted *p*-value, expression direction, and validation status were assessed. In instances where multiple probes corresponded to the same gene symbol, the probe with the highest mean normalized expression intensity across all samples in the validation comparison was selected, irrespective of disease group and differential expression statistics. This criterion, independent of outcomes, was employed to prevent selection bias associated with choosing probes based on adjusted *p*-values. The corresponding GEO2R logFC and Benjamini–Hochberg-adjusted *p*-value for the selected probe were subsequently used for validation. A gene was deemed statistically validated if it exhibited a positive logFC and an adjusted *p*-value of less than 0.05. Genes with a positive logFC but an adjusted *p*-value of 0.05 or greater were considered directionally concordant but not statistically validated. Conversely, genes displaying an expression direction contrary to that observed in the discovery dataset were deemed directionally inconsistent.

### 4.9. Mechanism Model Construction

The conceptual framework was developed exclusively to integrate transcriptomic, enrichment, and network findings with previously established biological pathways. It was not designed to represent a proven causal mechanism. As direct measurements of ferroptosis were absent in the analyzed datasets, associations with ferroptosis-related processes were regarded as hypothesis-generating.

### 4.10. Statistical Analysis and Visualization

Differential expression was defined using an adjusted *p*-value < 0.05, with adjusted *p*-values employed to control for multiple testing. Volcano plots, Venn diagrams, enrichment bar plots, PPI networks, hub-gene networks, and validation tables were generated using GEO2R, STRING, Cytoscape, g:Profiler, Microsoft Excel 365 (Microsoft Corporation, Redmond, WA, USA), and Python Colaboratory using Python version 3.10.3 where appropriate. Enrichment results were ranked by −log10-adjusted *p*-value. All analyses were conducted using publicly available datasets; thus, no additional ethical approval was required [20].

## 5. Conclusions

This study identified a transcriptional intersection between differential gene expression associated with active tuberculosis (TB) and a ferroptosis-associated gene set derived from FerrDb. Among the 1015 differentially expressed genes (DEGs) identified in the comparison between active and latent TB, 94 intersected with the ferroptosis-associated gene set. These genes were enriched in immune and stress-response processes, including innate immune signaling, neutrophil-associated responses, cytokine signaling, glutathione metabolism, and antimicrobial metal sequestration. Network analysis prioritized 10 predominantly immune–inflammatory hub genes: IL1B, TLR4, CXCL10, MMP9, CYBB, MPO, CD36, LCN2, S100A8, and LTF. External validation demonstrated heterogeneous reproducibility across datasets. LCN2 and LTF exhibited the strongest evidence, with significant positive differential expression in both independent validation datasets. IL1B, TLR4, MMP9, and MPO were statistically significant in GSE62525 and showed concordant positive but nonsignificant expression in GSE28623. CXCL10, CYBB, and CD36 exhibited positive but nonsignificant expression in both datasets, whereas S100A8 showed dataset-dependent expression. Importantly, these transcriptomic findings do not demonstrate ferroptotic cell death or establish a causal ferroptosis mechanism in active TB. Instead, they identify immune–inflammatory transcriptional changes that intersect with genes previously associated with ferroptosis, providing candidate genes and biological processes for further investigation. Future experimental studies incorporating direct measurements of lipid peroxidation, iron homeostasis, GPX4/GSH status, and ferroptosis-specific pharmacological rescue will be required to determine whether ferroptosis contributes mechanistically to TB-associated host responses and disease progression.

## Figures and Tables

**Table 1:** External validation of the 10 hub genes after outcome-independent probe selection.

Gene	GSE28623 Probe	logFC	adj. *p*-Value	GSE28623 Status	GSE62525 Probe	logFC	adj. *p*-Value	GSE62525 Status
*IL1B*	13475	0.598	0.2152	Concordant, not significant	PH_hs_0007166	1.970	4.82 × 10^−7^	Validated
*TLR4*	17810	0.870	0.0896	Concordant, not significant	PH_hs_0048721	1.050	7.88 × 10^−6^	Validated
*CXCL10*	12477	1.073	0.0912	Concordant, not significant	PH_hs_0024961	0.132	0.818	Concordant, not significant
*MMP9*	14852	0.933	0.0544	Concordant, not significant	PH_hs_0009143	2.410	0.00139	Validated
*CYBB*	35690	0.158	0.7473	Concordant, not significant	PH_hs_0004967	0.341	0.116	Concordant, not significant
*MPO*	38514	0.583	0.2608	Concordant, not significant	PH_hs_0026335	1.120	0.0157	Validated
*CD36*	35942	0.814	0.1079	Concordant, not significant	PH_hs_0044704	0.470	0.141	Concordant, not significant
*LCN2*	33122	1.153	0.0208	Validated	PH_hs_0011986	1.160	0.0119	Validated
*S100A8*	28674	1.703	0.00273	Validated	PH_hs_0045829	−0.0304	0.750	Directionally inconsistent
*LTF*	386	1.261	0.0407	Validated	PH_hs_0025606	1.270	0.000550	Validated

## Data Availability

The datasets analyzed in this study are publicly available in the Gene Expression Omnibus database under accession numbers GSE37250, GSE28623, and GSE62525. The processed results generated during this study are included in the manuscript and Supplementary Materials, and additional analysis files are available from the corresponding author upon reasonable request.

## Supplementary Materials

The following supporting information can be downloaded at: https://www.mdpi.com/article/10.3390/ijms27177757/s1.
