# Supplementary material for: Immune–Inflammatory Hub Genes Intersecting with a Ferroptosis-Associated Gene Set in Active Tuberculosis: A Multi-Dataset Bioinformatics Study
Source: Int J Mol Sci. 2026 Aug 29;27(17):7757. doi: 10.3390/ijms27177757 (PMC13565939; doi:10.3390/ijms27177757)
Supplement: Supplementary file 1 [file ijms-27-07757-s001.zip › ijms-4484555-supplementary.pdf]

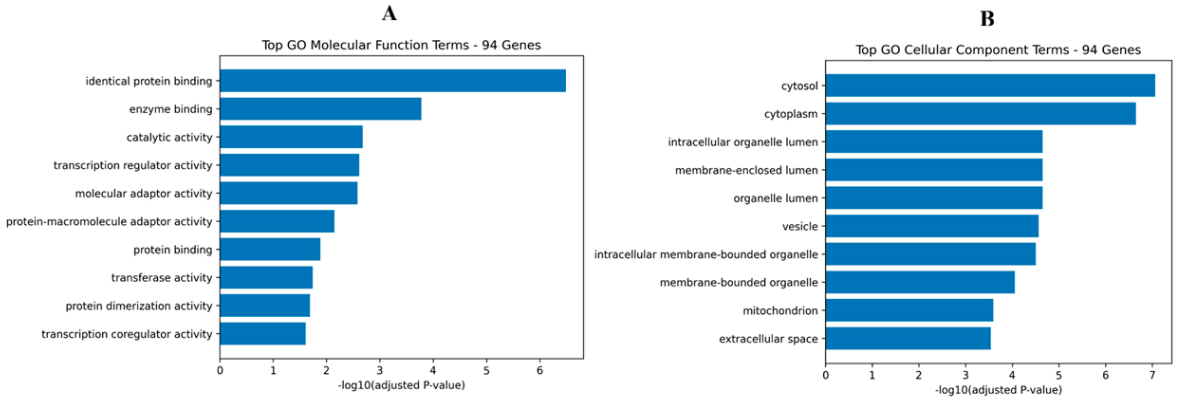

Supplementary Figure S1 presents the Gene Ontology (GO) molecular function and cellular component enrichment analysis of 94 ferroptosis-related differentially expressed genes (DEGs). The horizontal bar plots illustrate the top enriched GO terms, ranked by the −log10 adjusted P-value. Panel (A) reveals that the GO molecular function enrichment analysis identified significant associations of these DEGs with identical protein binding, enzyme binding, catalytic activity, transcription regulator activity, molecular adaptor activity, protein binding, transferase activity, protein dimerization activity, and transcription coregulator activity. Panel (B) demonstrates that the GO cellular component enrichment analysis localized these genes predominantly to the cytosol, cytoplasm, intracellular organelle lumen, membrane-enclosed lumen, organelle lumen, vesicles, intracellular membrane-bounded organelles, mitochondria, and extracellular space. These results underscore the role of the 94 ferroptosis-related DEGs in protein interaction, regulatory activity, intracellular stress responses, vesicle-associated processes, and mitochondrial-related cellular functions.

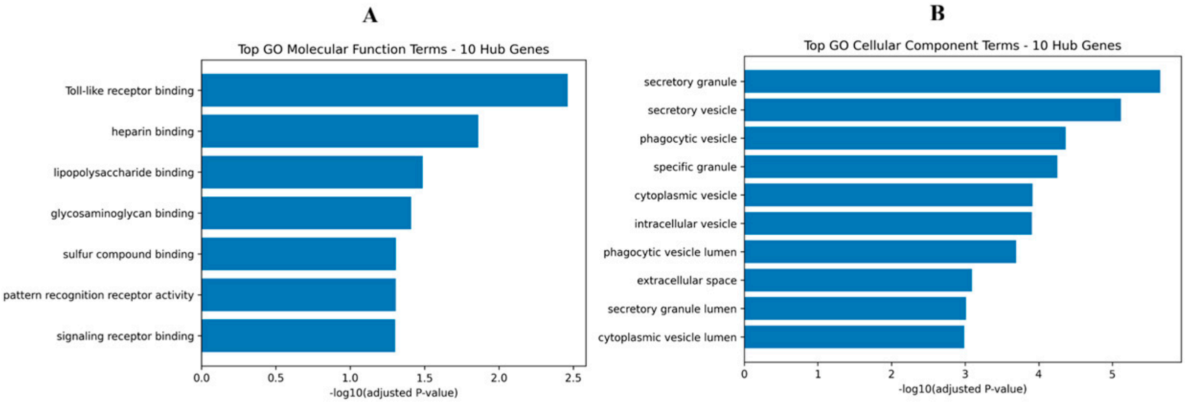

Supplementary Figure S2 presents the Gene Ontology (GO) molecular function and cellular component enrichment analysis of the 10 hub genes. The horizontal bar plots illustrate the top enriched GO terms, ranked by the −log10 adjusted P-value. Panel (A)

reveals that the GO molecular function enrichment of the 10 hub genes is significantly associated with Toll-like receptor binding, heparin binding, lipopolysaccharide binding, glycosaminoglycan binding, sulfur compound binding, pattern recognition receptor activity, and signaling receptor binding. Panel (B) demonstrates that the GO cellular component enrichment predominantly indicates localization in secretory granules, secretory vesicles, phagocytic vesicles, specific granules, cytoplasmic vesicles, intracellular vesicles, phagocytic vesicle lumen, extracellular space, secretory granule lumen, and cytoplasmic vesicle lumen. These findings substantiate the involvement of the hub genes in innate immune recognition, bacterial response, secretory/phagocytic activity, and inflammatory host-defense mechanisms in active tuberculosis.

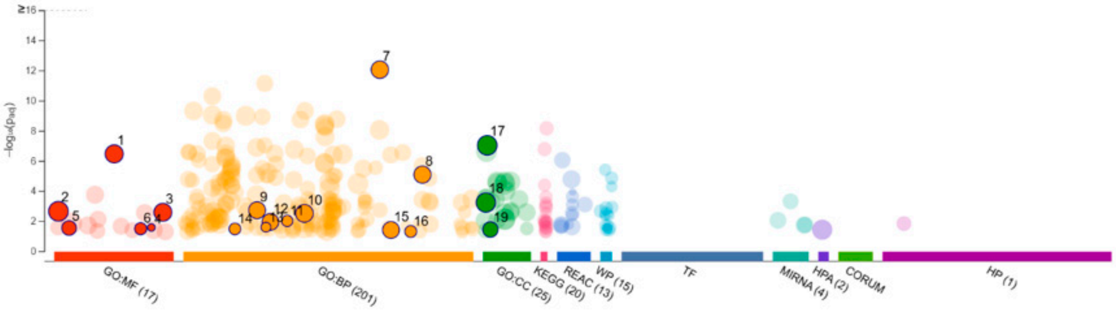

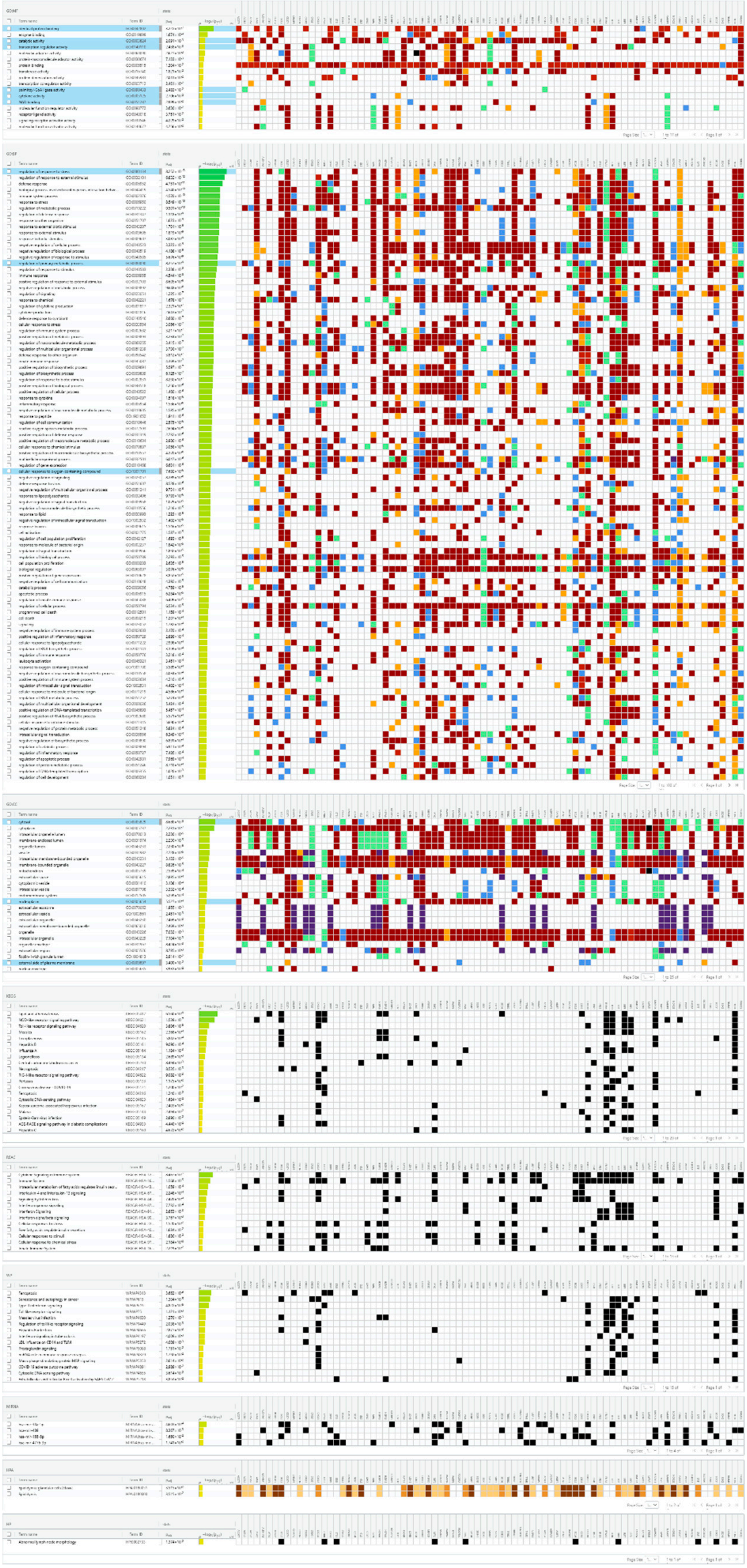

Supplementary Figure S3. Complete g:Profiler enrichment output for the 94 ferroptosis-related DEGs. Complete g:Profiler enrichment overview and gene-term intersection matrix for the 94 ferroptosis-related differentially expressed genes identified from the active TB versus latent TB comparison. The output includes enriched GO molecular function, GO biological process, GO cellular component, KEGG, Reactome, WikiPathways, Human Protein Atlas, and CORUM terms. The enrichment profile supports the involvement of the 94 ferroptosis-related DEGs in immune response, stress response, defense response, cellular metabolism, intracellular localization, and ferroptosis-associated biological processes.

Supplementary Table S3. Functional enrichment results for the 94 ferroptosis-associated intersection genes obtained using g:Profiler. The table presents selected significantly enriched Gene Ontology terms from the Molecular Function (GO:MF), Biological Process (GO:BP), and Cellular Component (GO:CC) categories. For each enriched term, the source, Gene Ontology term identifier, term name, and adjusted *p*-value are shown. Terms with an adjusted $p < 0.05$ were considered statistically significant.

ID	Source	Term ID	Term Name	p_{adj} (query_1)
1	GO:MF	GO:0042802	identical protein binding	3.222×10^{-7}
2	GO:MF	GO:0003824	catalytic activity	2.091×10^{-3}
3	GO:MF	GO:0140110	transcription regulator activity	2.438×10^{-3}
4	GO:MF	GO:0090433	palmitoyl-CoA ligase activity	2.483×10^{-2}
5	GO:MF	GO:0005125	cytokine activity	2.706×10^{-2}
6	GO:MF	GO:0051287	NAD binding	2.999×10^{-2}
7	GO:BP	GO:0080134	regulation of response to stress	8.252×10^{-13}
8	GO:BP	GO:1901701	cellular response to oxygen-containing compound	7.692×10^{-6}
9	GO:BP	GO:0030097	hemopoiesis	1.783×10^{-3}
10	GO:BP	GO:0044281	small molecule metabolic process	2.882×10^{-3}
11	GO:BP	GO:0036037	CD8-positive, alpha-beta T cell activation	9.203×10^{-3}
12	GO:BP	GO:0032880	regulation of protein localization	1.066×10^{-2}
13	GO:BP	GO:0032306	regulation of prostaglandin secretion	2.376×10^{-2}
14	GO:BP	GO:0014002	astrocyte development	3.094×10^{-2}
15	GO:BP	GO:0098657	import into cell	3.648×10^{-2}
16	GO:BP	GO:1900225	regulation of NLRP3 inflammasome complex asse...	4.587×10^{-2}
17	GO:CC	GO:0005829	cytosol	8.680×10^{-8}
18	GO:CC	GO:0005654	nucleoplasm	5.527×10^{-4}
19	GO:CC	GO:0009897	external side of plasma membrane	3.400×10^{-2}

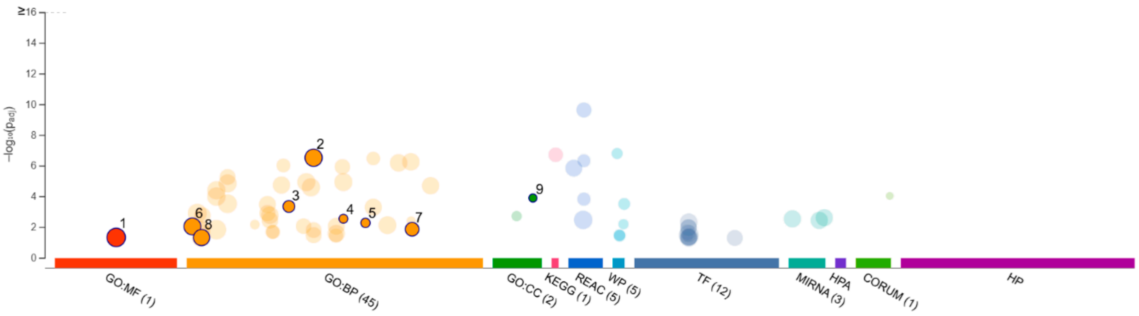

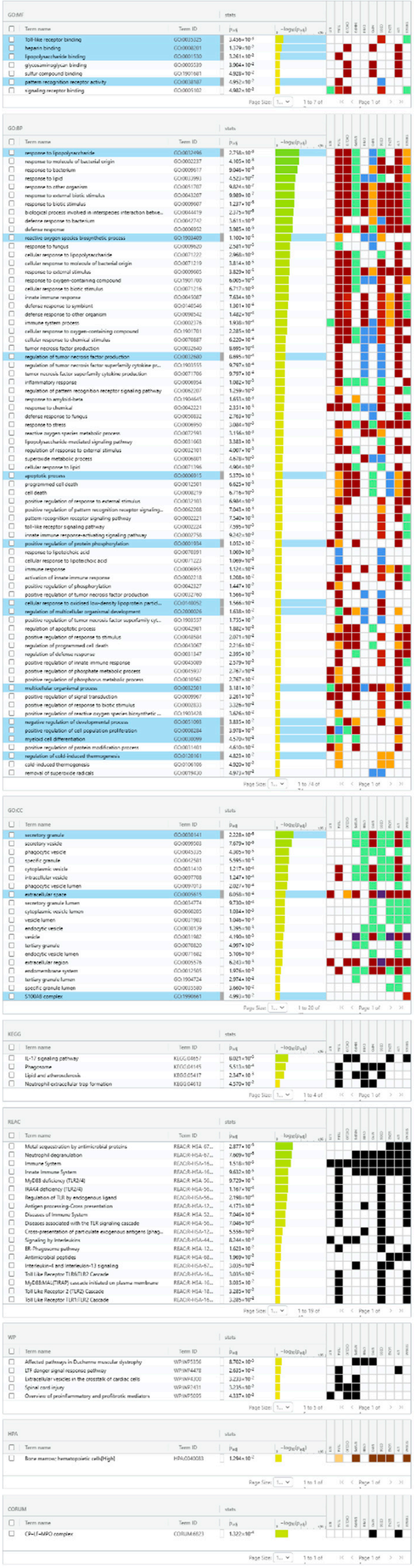
GO:MF
GO:BP
GO:CC
KEGG
REAC
WP
HPA
CORUM

Supplementary Figure S4. Complete g:Profiler enrichment output for the 10 hub genes. Complete g:Profiler enrichment overview and gene-term intersection matrix for the 10 prioritized hub genes. The output includes enriched GO, KEGG, Reactome, WikiPathways, Human Protein Atlas, and CORUM terms. The hub genes showed enrichment in biological processes and pathways related to bacterial response, lipopolysaccharide response, innate immune recognition, neutrophil degranulation, secretory and phagocytic vesicles, Toll-like receptor binding, and inflammatory host-defense mechanisms.

Supplementary Table S4. Functional enrichment results for the 10 hub genes obtained using g:Profiler. The table presents the significantly enriched Gene Ontology terms identified for the hub-gene set, including Molecular Function (GO:MF), Biological Process (GO:BP), and Cellular Component (GO:CC) categories. For each term, the Gene Ontology identifier, term name, and adjusted *p*-value are reported. Terms with an adjusted $p < 0.05$ were considered statistically significant.

ID	Source	Term ID	Term Name	p_{adj} (query_1)
1	GO:MF	GO:0042802	identical protein binding	4.498×10^{-2}
2	GO:BP	GO:0045087	innate immune response	2.874×10^{-7}
3	GO:BP	GO:0035456	response to interferon-beta	4.305×10^{-4}
4	GO:BP	GO:0051715	cytolysis in another organism	2.726×10^{-3}
5	GO:BP	GO:0070106	interleukin-27-mediated signaling pathway	5.086×10^{-3}
6	GO:BP	GO:0001817	regulation of cytokine production	8.685×10^{-3}
7	GO:BP	GO:0140888	interferon-mediated signaling pathway	1.316×10^{-2}
8	GO:BP	GO:0002757	immune response-activating signaling pathway	4.597×10^{-2}
9	GO:CC	GO:0106139	symbiont cell surface	1.216×10^{-4}
